# Nanosecond pulsed electric field ablation as first-line curative therapy for hepatocellular carcinoma in high-risk locations a prospective multicenter

**DOI:** 10.1097/JS9.0000000000002361

**Published:** 2025-03-28

**Authors:** Min Xu, Wu Zhang, Danxia Xu, Gang Dong, Zhigang Ren, Tuerganaili Aji, Jiansong Ji, Qiyu Zhao, Jinhua Pan, Xinhua Chen, Tian’An Jiang

**Affiliations:** a Department of Ultrasound Medicine, The First Affiliated Hospital, School of Medicine, Zhejiang University, Hangzhou, China; b Shulan (Hangzhou) Hospital Affiliated to Zhejiang Shuren University, Shulan International Medical College, Hangzhou, China; c Department of Ultrasound, the First Affiliated Hospital of Zhengzhou University, Zhengzhou, China; d Department of Infectious Diseases, The First Affiliated Hospital of Zhengzhou University, Zhengzhou, China; e Department of Surgery, The First Affiliated Hospital of Xinjiang Medical University, Urumqi, China; f Key Laboratory of Imaging Diagnosis and Minimally Invasive Intervention Research, Lishui Central Hospital and Fifth Affiliated Hospital of Wenzhou Medical College, Lishui, China; g Department of Hepatobiliary and Pancreatic Surgery, The First Affiliated Hospital, School of Medicine, Zhejiang University, Hangzhou, China

**Keywords:** electroporation, hepatocellular carcinoma, minimally invasive procedures

## Abstract

**Background:**

Preclinical studies have shown that nanosecond pulsed electric field ablation (nsPEF) is a novel nonthermal ablation modality that can eradicate tumors near critical structures. We conducted the prospective multicenter trial to investigate the efficacy and safety of nsPEF for hepatocellular carcinoma (HCC) in high-risk locations.

**Materials and methods:**

This study was conducted at five hospitals in China. Patients with HCC fulfilling the Milan criteria and located immediately adjacent to (<0.5 cm) the portal vein, hepatic vein, diaphragm, gastrointestinal tract, liver capsule, or gallbladder were enrolled. The primary endpoint was the complete ablation rate at 1 month, and adverse events. The secondary endpoints included local tumor progression (LTP), recurrence-free survival (RFS), and overall survival.

**Results:**

From March 2020 to June 2022, 192 patients were enrolled (148 males [77.1%]; median age 58.5 years [interquartile range, 51.0–66.0 years]). The median follow-up duration was 33.5 months. The technical success rate was 99.5%. Complete ablation was achieved in 91.7% of the 217 tumors. Complete ablation rates at 1 month were significantly higher in tumors <2 cm vs. ≥2 cm (90.1% vs. 71.7%, *P* = 0.002). The estimated 1-, 2- and 3-year cumulative incidences of LTP were 9.8%, 13.8%, and 15.7%, respectively. The maximum tumor diameter (hazard ratio [HR] = 2.62, *P* = 0.014) and age (HR = 0.42, *P* = 0.026) were independent predictive factors for LTP. The RFS rates at 1-, 2- and 3-year were 72.2%, 51.7%, and 43.5%, respectively. No periprocedural thermal damage was observed. Grade ≥3 treatment-related adverse events occurred in nine (5.6%) patients.

**Conclusion:**

To our knowledge, this was the first prospective trial demonstrating that nsPEF was effective and relatively safe for HCC in high-risk locations, and may serve as an alternative therapeutic option for HCC suboptimal for thermal ablation.

**HIGHLIGHTS**

- The nanosecond pulsed electric field ablation (nsPEF) demonstrates high feasibility for treating hepatocellular carcinoma (HCC) in high-risk locations, with a 99.5% procedural success rate.
- Complete ablation was achieved in 91.7% of the 217 tumors treated.
- The nsPEF maybe an alternative therapeutic option for HCC suboptimal for thermal ablation.

## Introduction

Liver cancer is the third leading cause of cancer-related death worldwide, and hepatocellular carcinoma (HCC) accounts for 80% of all liver cancer^[^1–4^]^. Early-stage HCCs can be cured with surgical resection or liver transplantation, but the majority of patients are not candidates for surgery and are instead treated with thermal ablation techniques, such as radiofrequency ablation (RFA) or microwave ablation (MWA)^[^5,6^]^. Although thermal ablation has been shown to provide good local disease control for small, localized HCCs, its suitability for challenging tumor locations is limited^[^3,7^]^. Thermal damage to adjacent structures (e.g. large blood vessels, bile ducts, and intestines) can result in serious complications^[^8,9^]^. Moreover, the thermal ablation of tumors adjacent to large vessels is associated with a higher incidence of incomplete eradication (the heat-sink effect)^[^10^]^.

High-voltage electrical pulses have been applied as a novel nonthermal ablation modality^[^11,12^]^. These electrical pulses permeabilize the cell membrane, causing cell death. Unlike thermal ablation, this treatment does not affect the extracellular matrix, and thus maintains the integrity of blood vessels and bile ducts. In addition, its efficacy is not impeded by the heat-sink effect^[^13,14^]^. Traditional pulsed field ablation (irreversible electroporation [IRE]) is based on microsecond-scale pulses. Recently, another electrical engineering technology has been developed, called nanosecond pulsed electric field ablation (nsPEF).

The durations of nsPEF are shorter than the plasma membrane charge times, enabling not only membrane penetration but also action on organelles such as the endoplasmic reticulum, mitochondria, and nucleus^[^15^]^. Subsequent biological responses include calcium mobilization^[^16^]^, reactive oxygen species production^[^17^]^, and the rapid externalization of phosphatidylserine^[^18^]^, which induce cell death. Moreover, compared with IRE, nsPEF can minimize their efficacy to stimulate neurons and cardiomyocytes, and reduce intractable side effects, including involuntary muscle contractions and pain^[^19,20^]^. The nsPEF has shown promising therapeutic prospects for HCC in both cells and animal experiments (Supplemental Digital Content 1, available at: http://links.lww.com/JS9/E39). Here, we conducted the prospective multicenter trial to evaluate the safety and efficacy of nsPEF in patients with high-risk locations HCC.

## Materials and methods

### Study design and participants

This multicenter prospective study was conducted at five hospitals in China. Patients diagnosed with HCC as per the American Association for the Study of Liver Diseases guidelines and determined by a multidisciplinary tumor board to be suboptimal location for thermal ablation were included. The high-risk locations were defined as a maximum distance of 5 mm from the portal vein (the right/left portal vein or the second order branch), hepatic vein (the base of hepatic vein or the inferior vena cava), diaphragm, gastrointestinal tract, liver capsule, gallbladder, or bile duct (right/left bile duct)^[^21,22^]^. The other inclusion criteria were: age >18 years, fulfillment of the Milan criteria (single tumor up to 5 cm or 2–3 tumor nodules up to 3 cm in size, without vascular invasion), Eastern Cooperative Oncology Group score ≤2, Child–Pugh class A or B, and adequate hematological and organ function. Patients with uncontrolled ascites, hepatic encephalopathy, histories of epilepsy or ventricular cardiac arrhythmia, implanted stimulation devices, and/or other malignant tumors were excluded (details are provided in the study protocol).

All participants provided written informed consent to study enrollment. The study was approved by the institutional review boards of all participating hospitals and conducted in accordance with the Declaration of Helsinki. It has been registered at ClinicalTrials.gov and is reported in line with the STROCSS criteria (Supplemental Digital Content 1, available at: http://links.lww.com/JS9/E39)^[^23^]^.

### The nsPEF procedure

All included patients underwent radiological assessments, including abdominal contrast-enhanced magnetic resonance imaging (MRI) or contrast-enhanced computed tomography (CT) when MRI was contraindicated. Three-dimensional tumor measurements were used to determine the required electrode number and configuration. The nsPEF procedures were conducted in close accordance with the recommendations set forth by the system vendor and in accordance with standard operating procedures established by experienced interventional radiologists of all participating hospitals. All nsPEF procedures were performed with the patients under general anesthesia with complete muscular relaxation. With real-time US or CT guidance, 2–6 19-gauge unipolar nsPEF electrodes were inserted parallelly and percutaneously according to the treatment plan, with the aim of achieving an interelectrode distance of 10–25 mm and active tip length of 20 mm. The nsPEF device (Ruidi Biotechnology Co., Ltd., Hangzhou, Zhejiang, China) was used for treatment. Given the unique ablation principle of nsPEF treatment, the artificial ascites technique was not used to separate a tumor from an important structure in any patient. Some advanced technologies such as artificial pleural effusion, three-dimensional visualization, and multimodal image fusion navigation were used to improve visibility of target tumors, when US guided. After confirmation that the electrodes were placed correctly, a 5-kV test pulse was delivered. The adequacy of conductivity was confirmed, and nsPEF treatment was conducted with 800 pulses of 25–30 kV/cm and a pulse length of 300 ns. After completion of the pulse applications, CT or contrast-enhanced ultrasound (CEUS) was performed to confirm sufficient ablation, which was defined as an ablation zone that included the entire target tumor. If the extent of the ablation zone was suspected insufficient, additional cycles of energy depositions for overlapped ablations were performed, preferably after electrode pullbacks (from 1 to 2 cm partial withdrawal of needles along the axis of the initial puncture) and/or partial or complete electrodes reinsertion (in different axis from initial puncture). Further details of the treatment method are provided in Supplemental Digital Content 1, available at: http://links.lww.com/JS9/E39.

### Follow-up

On the day after nsPEF treatment, routine laboratory studies, electrocardiography, and CEUS were performed to identify any procedure-related complications. Contrast-enhanced MRI examinations were performed 1 month after the procedures to evaluate their efficacy. Complete ablation was defined by the complete nonenhancement of the treated tumor, and incomplete ablation was defined by the presence of residual tumor on contrast-enhanced MRI/CT^[^24^]^. In the event of incomplete ablation, an additional nsPEF procedure was conducted using the same technique. If the residual tumor remained viable after the second session, the patient was excluded from the trial and referred for other treatment. In cases of confirmed treatment efficacy, patients underwent clinical, biological, and radiological examinations every 3 months for the first 2 years and every 6 months thereafter.

### Endpoints

Endpoints and definitions were specified using the consensus guidelines for image-guided tumor ablation^[^24^]^. Because this was the first-in-human trial, the primary endpoints were the complete ablation rate at 1 month, and adverse events (AEs). AEs were graded according to the National Cancer Institute Common Terminology Criteria (version 5.0). The secondary endpoints were technical success, technique efficacy, local tumor progression (LTP), recurrence-free survival (RFS), and overall survival (OS) (Supplemental Digital Content 1, available at: http://links.lww.com/JS9/E39). Technical success was identified as a tumor treated in accordance with protocol and covered completely by the ablation zone. Technique efficacy was defined as radiologic complete ablation achieved after as many as two iterative nsPEF procedures. Tumor recurrence after ablation was further classified into three categories: LTP, intrahepatic distant recurrence, and extrahepatic metastasis. A radiological assessment training session was held at each participating center before the commencement of the study to reach consensus on the imaging criteria and reduce the risk of bias.

### Sample size

As this was a pilot study, no existing data on the complete ablation rate achieved with the nsPEF treatment were available. Complete ablation rates of 77.3%–86.0% after initial IRE (with a traditional microsecond duration) treatments in patients with HCC have been reported^[^25,26^]^. We thus hypothesized that we would achieve complete ablation of 80% of the tumors in high-risk locations treated with nsPEFs by the 1-month follow-up time point. For this single-arm objective performance criteria study, employing 80% power and a 2.5% one-sided *α* threshold, we determined that the enrollment of 160 patients would be required. Assuming a 20% rate of withdrawal or loss to follow-up, we sought to enroll 192 participants.

### Statistical analysis

Normally and non-normally distributed continuous variables are expressed as means ± standard deviations and medians with interquartile ranges (IQRs), respectively. Categorical data are expressed as numbers and percentages. To determine the factors significantly associated with the initial complete ablation, binary logistic regression analysis was performed. The survival analyses were performed using the Kaplan–Meier method. Multivariate Cox proportional-hazards regression was used to identify prognostic factors of LTP, RFS, and OS. A *P* value of less than 0.1 at univariable analysis was included for multivariable analysis. *P* values of less than 0.05 were considered to indicate significant difference. The data were analyzed using SPSS software (version 22.0; IBM Corporation, Armonk, NY, USA).

## Results

### Participant characteristics

**Figure 1.**
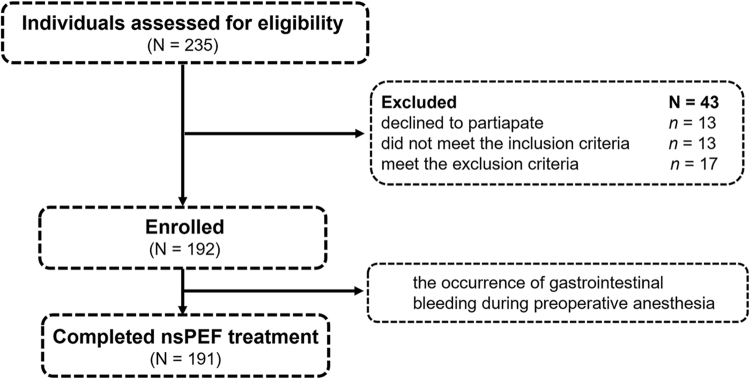
The flowchart. nsPEF, nanosecond pulsed electric field.

Individuals were enrolled between 12 March 2020, and 28 June 2022. Of the 235 participants screened, 192 were finally included in the study (Fig. 1). Forty-three participants were excluded: 13 participants were excluded for refusal to participate in the study; 13 participants did not meet the inclusion criteria; and 17 participants meet the exclusion criteria. The median follow-up duration at the time of final analysis (1 June 2024) was 33.5 (IQR, 24.0–50.6) months. In total, 191 patients with 217 tumors completed the nsPEF treatment. One procedure was terminated before completion due to the occurrence of gastrointestinal bleeding during anesthesia; data from this patient were included only in the safety analysis.

The median patient age was 58.5 (IQR, 51–66) years, 77.1% of the patients were male, and 85.4% had Child–Pugh liver classifications of A. The main etiology of HCC was hepatitis B virus infection (81.3%), and more than half of the patients had cirrhosis. The median maximum tumor diameter was 1.6 (IQR, 1.3–2.0) cm. The tumors were most commonly located in Couinaud segment VIII (19.2%), followed by segments Ⅶ (17.4%), Ⅴ (16.4%), and Ⅳ (16.4%). All tumors were immediately adjacent or attached to high-risk structures, consisting mainly of the portal vein (37.9%), liver capsule (22.4%), hepatic vein (19.6%), diaphragm (6.8%), and gastrointestinal tract (6.8%). The patients’ baseline demographic and clinical characteristics are summarized in Table 1.

**Table 1.** Baseline patient characteristics.

Characteristic	*n* (%) or median (IQR)
Age, years	58.5 (51–66)
Sex	
Male	148 (77.1)
Female	44 (22.9)
Etiology	
HBV	156 (81.3)
HCV	10 (5.2)
Unknown or other	26 (13.5)
Child–Pugh class	
A	164 (85.4)
B	28 (14.6)
ALBI grade	
1	116 (60.4)
2	76 (39.6)
ECOG performance status	
0	135 (70.3)
1	42 (21.9)
2	15 (7.8)
Cirrhosis	
Absent	42 (21.9)
Present	150 (78.1)
Portal hypertension	
Absent	109 (56.8)
Present	83 (43.2)
α-Fetoprotein level, ng/mL	7.7 (2.9–49.2)
Albumin, g/L	40.7 (26.7–73.9)
TBil, μmol/L	14.4 (10.0–21.7)
Platelet count, 10^9^/L	116.0 (79.3–164.5)
Prothrombin time, seconds	12.2 (11.4–13.2)
Previous local therapy	
Absent	71 (37.0)
Present	121 (63.0)
Resection	74 (38.5)
Ablation	68 (35.4)
TACE	47 (24.5)

ALBI, albumin-bilirubin; ECOG, Eastern Cooperative Oncology Group; HBV, hepatitis B virus; HCV, hepatitis C virus; IQR, interquartile range; TACE, transcatheter arterial chemoembolization; TBil, total bilirubin.

### Procedure characteristics

**Figure 2.**
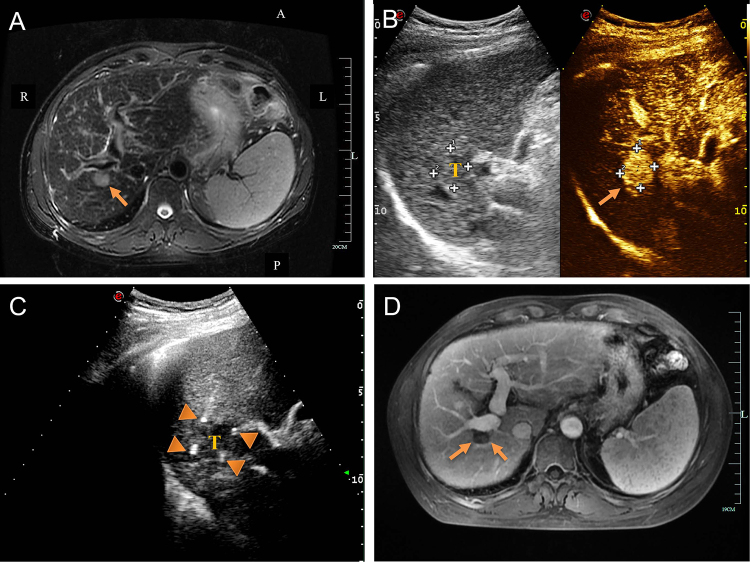
Ultrasound-guided nanosecond pulsed electric field (nsPEF) procedure for HCC adjacent to portal vein. (A) Pre-ablation MRI revealed a hepatocellular carcinoma adjacent to portal vein (arrow). (B) Contrast-enhanced ultrasound showed an enhancement tumor located in close contact with portal vein (arrow). (C) Ultrasound-guided nsPEF with a two-electrode configuration, and the head and tail end of the active tip of the electrodes can have punctuated enhancements (triangle). (D) The contrast-enhanced MRI image revealed no enhancement after ablation (arrow).

**Figure 3.**
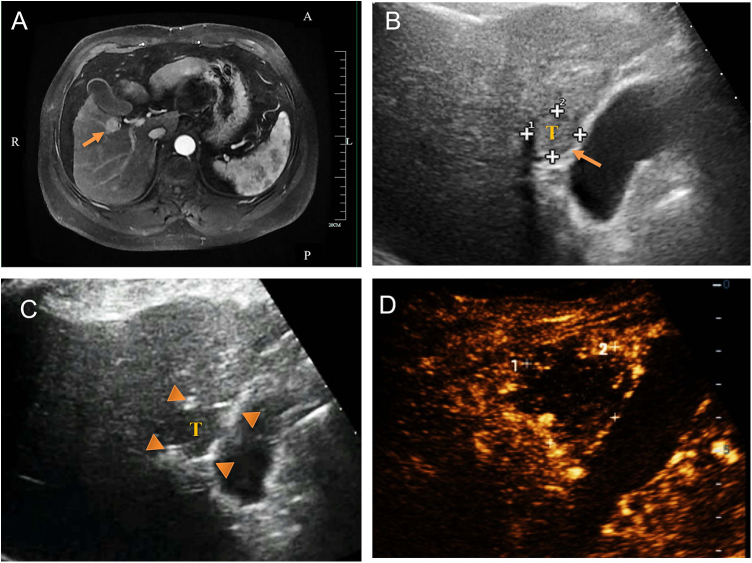
Ultrasound-guided nanosecond pulsed electric field (nsPEF) procedure for HCC adjacent to gallbladder. (A) Pre-ablation contrast-enhanced MRI revealed a hepatocellular carcinoma adjacent to gallbladder (arrow). (B) Ultrasound showed a hypoecho tumor located in close contact with gallbladder (arrow). (C) Two 19-gauge unipolar nsPEF electrodes (triangle) were inserted parallelly and percutaneously around the tumor. (D) The contrast-enhanced ultrasound image revealed no enhancement after ablation.

The nsPEF treatment was technically successful in 191 (99.5%) of 192 cases. Initial procedures were performed to treat one (*n* = 167), two (*n* = 23), and three (*n* = 2) tumors per participant (Table 2). Two to four electrodes were placed with US (91.8%) and CT (8.2%) guidance. Some advanced technologies such as artificial pleural effusion (*n* = 3), three-dimensional visualization (*n* = 2), and multimodal image fusion navigation (*n* = 5) were used to improve US visibility of target tumors. The electrodes were placed at 10–25 mm (median, 15 mm) intervals. The median ablation time was 49.2 (IQR, 35.3–75.2) min and the median ablation zone size was 2.9 (IQR, 1.8–3.9) cm. Given the unique ablation principle of nsPEF treatment, the artificial ascites technique was not used to separate a tumor from an important structure in any patient. The median hospital stay was 3 (IQR, 2–11) days. Examples of successful tumor eradication with nsPEF are provided in Figures 2 and 3.

**Table 2.** Tumor and procedure characteristics.

Characteristic	*n* (%) or median (IQR)
Tumor diameter, mm	16 (13–20)
Tumor number	219
1	167 (87.0)
2	23 (12.1)
3	2 (1.0)
No. of procedures	208
Initial procedure	192 (92.3)
Second procedure for incomplete ablation	16 (7.7)
Liver segment tumor distribution	
I	4 (1.8)
II	16 (7.3)
III	17 (7.8)
IV	36 (16.4)
V	36 (16.4)
VI	30 (13.7)
VII	38 (17.4)
VIII	42 (19.2)
Adjacent/attached risk structures	
Portal vein	83 (37.9)
Hepatic vein	43 (19.6)
Gallbladder	14 (6.4)
Diaphragm	15 (6.8)
Liver capsule	49 (22.4)
Gastrointestinal tract	15 (6.8)
No. of electrodes used	2 (2–4)
Maximum voltage, kV^a^	30 (25–30)
Electrode interval, mm^a^	15 (10–25)
Length of active tip, mm	20
Imaging guidance	
Ultrasound	191 (91.8)
CT	17 (8.2)
Ablation zone size, mm	29 (18–39)
Hospital stays, days	3 (2–11)
Ablation time, min	49.2 (35.3–75.2)

CT, computerized tomography; IQR, interquartile range.

^a^ Numbers in parentheses are the range.

### Safety

No patient died within 90 days after nsPEF treatment. No periprocedural cardiac arrhythmia or thermal damage was observed. Overall, 259 AEs (93.8% grade 1) occurred in 159 of the 192 participants. Common AEs were transient increases in the aminotransferase level (55.6%), fever (7.3%), mild abdominal pain (5.8%), hypertension (5.3%), and incision pain (3.1%). The aspartate aminotransferase (AST) and alanine aminotransferase (ALT) levels increased sharply within 24 h after nsPEF treatment in 143 (67.5%) patients, and had returned to normal level at 1 month after the procedure in 89 (84.8%) of these patients. Sixteen (6.2%) patients experienced grade 3–4 AEs, nine of which were treatment related. Two patients developed postoperative pleural effusion requiring drainage (grade 3), one patient with HCC close to the gallbladder developed mild cholecystitis (grade 3) and recovered after drug treatment, three patients developed small subcapsular hematomas visible on immediate post-interventional CT or CEUS images with no sign of active bleeding (grade 3), one patient developed aspiration pneumonia requiring intensive care unit admission (grade 4), and two patients had upper gastrointestinal bleeding and recovered under medical treatment (grade 4). The grading of all treatment-related AEs is presented in (Table 3), and details of AEs not related to treatment are provided in Supplemental Digital Content 1, available at: http://links.lww.com/JS9/E39.

**Table 3.** Treatment-related adverse events.

Event	All	Grades 1–2	Grade 3	Grade 4
Fever	19 (7.8)	19 (7.8)	0 (0.0)	0 (0.0)
Abdominal pain	15 (6.1)	15 (6.1)	0 (0.0)	0 (0.0)
Fatigue	2 (0.8)	2 (0.8)	0 (0.0)	0 (0.0)
Abdominal distension	2 (0.8)	2 (0.8)	0 (0.0)	0 (0.0)
Hypertension	9 (3.7)	9 (3.7)	0 (0.0)	0 (0.0)
Hydrothorax	2 (0.8)	0 (0.0)	2 (0.8)	0 (0.0)
Chest distress	2 (0.8)	2 (0.8)	0 (0.0)	0 (0.0)
Nausea, vomiting	3 (1.2)	3 (1.2)	0 (0.0)	0 (0.0)
Incision pain	8 (3.3)	8 (3.3)	0 (0.0)	0 (0.0)
Cholecystitis	1 (0.4)	0 (0.0)	1 (0.4)	0 (0.0)
Rash	4 (1.6)	4 (1.6)	0 (0.0)	0 (0.0)
Aspiration pneumonia	1 (0.4)	0 (0.0)	0 (0.0)	1 (0.4)
Needle bleeding	3 (1.2)	0 (0.0)	3 (1.2)	0 (0.0)
Gastrointestinal bleeding	2 (0.8)	0 (0.0)	0 (0.0)	2 (0.8)
Tracheal mucosa damage from intubation	2 (0.8)	2 (0.8)	0 (0.0)	0 (0.0)
Lower-limb vein thrombosis	1 (0.4)	1 (0.4)	0 (0.0)	0 (0.0)
Aminotransferase elevation	143 (58.0)	143 (58.0)	0 (0.0)	0 (0.0)
D-dimer elevation	4 (1.6)	4 (1.6)	0 (0.0)	0 (0.0)
Increased white blood cell count	4 (1.6)	4 (1.6)	0 (0.0)	0 (0.0)
Decreased white blood cell count	6 (2.5)	6 (2.5)	0 (0.0)	0 (0.0)
Decreased platelet count	6 (2.5)	3 (1.2)	3 (1.2)	0 (0.0)
Pneumonitis	2 (0.8)	0 (0.0)	2 (0.8)	0 (0.0)
Anemia	4 (1.6)	3 (1.2)	1 (0.4)	0 (0.0)

Data are reported as *n* (%).

### Complete ablation rate

**Figure 4.**
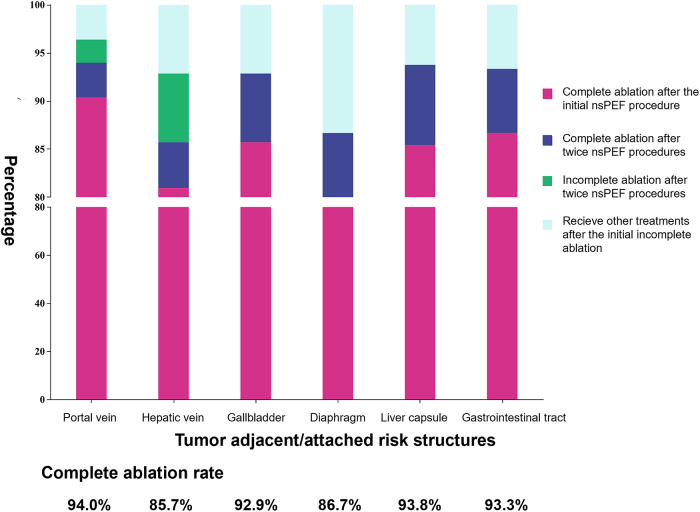
Complete ablation rates of tumors in high-risk locations treated by nsPEF. nsPEF, nanosecond pulsed electric field.

Of the 217 tumors treated, 187 were completely ablated after the initial nsPEF procedure. The complete ablation rate at 1 month was 86.2% (95% confidence interval [CI], 81.5%–90.8%]. Complete ablation rates at 1 month were significantly higher in tumors <2 vs. ≥ 2 cm (90.1% vs. 71.7%, *P* = 0.002). Second nsPEF procedures were performed for 18 incompletely ablated tumors; after these repeat procedures, complete ablation of the target tumor was achieved in 199 of the 217 tumors (91.7%; 95% CI, 88.0%–95.4%) (Fig. 4). The technique efficacy rate was 91.7%. After the initial nsPEF procedures, 12 participants were considered unsuitable for or refused to repeat nsPEF treatment, and their residual tumors were treated with TACE (*n* = 4), thermal ablation (*n* = 4), chemotherapy (*n* = 1), radiotherapy (*n* = 1), and surgery (*n* = 1); one patient was lost to follow-up. Multivariable logistic regression analysis revealed that the maximum tumor diameter (odds ratio, 3.57; 95% CI, 1.58–8.05) was the only independent predictive factor for initial complete ablation (Supplemental Digital Content 1, available at: http://links.lww.com/JS9/E39).

### LTP, RFS, and OS

**Figure 5.**
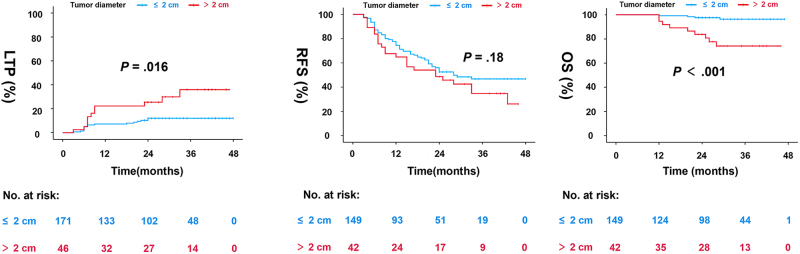
Kaplan–Meier curves for (A) LTP, (B) RFS, and (C) OS. LTP, local tumor progression; OS, overall survival; RFS, recurrence-free survival.

LTP occurred in 27 of 199 (13.6%, 95% CI, 8.8%–18.4%) HCCs in which nsPEF had been considered technically effective. The estimated 1-, 2-, and 3-year cumulative incidences of LTP were 9.8%, 13.8%, and 15.7%, respectively (Fig. 5). On multivariable Cox regression analysis, the age (hazard ratio [HR], 0.42; 95% CI, 0.19–0.90) and maximum tumor diameter (HR, 2.62; 95% CI, 1.21–5.65) were independent predictive factors for LTP. Other factors, including initial complete ablation, showed no significant predictive ability (Table 4).

**Table 4.** Predictive factors for LTP of nsPEF.

	Univariate		Multivariate	
Variable	HR (95% CI)	*P*	HR (95% CI)	*P*
Age >60 y	0.44 (0.20–0.95)	0.036	0.42 (0.19–0.90)	0.026
Male	1.28 (0.49–3.39)	0.616		
Multiple tumors	1.05 (0.42–2.60)	0.915		
Tumor diameter >2 cm	2.57 (1.19–5.55)	0.016	2.62 (1.21–5.65)	0.014
AFP >200 ng/mL	0.98 (0.34–2.83)	0.969		
Viral hepatitis	1.65 (0.39–6.96)	0.496		
Cirrhosis	3.78 (0.90–15.97)	0.070	4.22 (1.00–17.86)	0.051
PLT <100 × 10^9^/L	1.54 (0.72–3.27)	0.265		
ALB <35 g/L	0.90 (0.31–2.62)	0.853		
TBil >17.1 µmol/L	1.03 (0.46–2.29)	0.949		
Tumor adjacent to vessels	0.80 (0.38–1.71)	0.571		
Child-Pugh B	0.57 (0.14–2.42)	0.449		
CT guidance	1.08 (0.25–4.60)	0.914		
Initial complete ablation	1.86 (0.56–6.18)	0.311		

AFP, α-fetoprotein; ALB, albumin; CI, confidence interval; HR, hazard ratio; PLT, platelet; TBIL, total bilirubin.

The overall recurrence rate was 53.7% (95% CI, 45.9–61.5%). The median RFS duration was 28 (95% CI, 21.1–34.9) months. The RFS rates at 1-, 2-, and 3-year were 72.2%, 51.7%, and 43.5%, respectively (Fig. 5). The cumulative intrahepatic distant recurrence rates at 1-, 2-, and 3-year were 17.9%, 35.9%, and 41.0%, respectively, and the corresponding cumulative extrahepatic recurrence rates were 6.9%, 10.1%, and 14.7%, respectively. On multivariate analysis of data from all patients enrolled in the study, liver cirrhosis (HR, 2.39; 95% CI, 1.15–4.93) and previous treatment history (HR, 1.75; 95% CI, 1.10–2.80) emerged as factors associated significantly with poor RFS. Of the 80 patients with recurrence, 62 (77.5%) underwent subsequent antitumor therapies: 32 patients underwent thermal ablation, 21 underwent TACE, 3 underwent repeat surgical resection, 2 were given sorafenib, 2 underwent stereotactic body radiation therapy, and 2 underwent liver transplantation.

**Figure 6.**
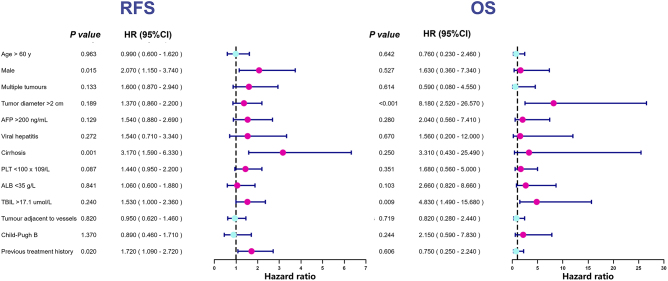
Forest plots of RFS and OS. AFP, α-fetoprotein; ALB, hemoglobin; CI, confidence interval; HR, hazard ratio; OS, overall survival; PLT, platelet; RFS, recurrence-free survival; TBIL, total bilirubin.

The overall mortality rate was 8% (95% CI, 3.8–12.3%). The median OS duration was not reached. The estimated OS rates at 1-, 2-, and 3-year were 98.1%, 94.4%, and 91.0%, respectively. The preoperative total bilirubin level (HR, 5.14; 95% CI, 1.58–16.72) and maximum tumor diameter (HR, 8.61; 95% CI, 2.64–28.03) were identified as independent prognostic factors for OS (Fig. 6).

## Discussion

In this multicenter study, we prospectively evaluated the use of nsPEF treatment in patients with early-stage HCC in high-risk locations who were suboptimal candidates for thermal ablation. We hypothesized that nsPEF would be an effective and safe means of treating tumors located near critical structures, such as vessels and hilar biliary ducts for central locations and the diaphragm and digestive tract for peripheral subcapsular locations. In these challenging cases, we achieved technical success and complete ablation rates exceeding 90%, with a moderate incidence of LTP at 2 years and low incidence of high-grade AEs.

The nsPEF is an emerging bioelectrical technology in cancer therapy. The nanosecond pulses with a high-intensity electric field change the permeability and electric properties of the plasma membrane and intracellular organelle membrane, which eventually result in apoptosis or apoptosis-like cell death^[^15^]^. In previous preclinical animal studies, nsPEF has been tested in various solid tumors, including HCC, melanoma, pancreatic cancer, basal cell carcinoma, and squamous cell carcinoma. In clinical trials, nsPEF proved to be an effective therapy against basal cell carcinoma^[^27^]^. By far, HCC is one of the most-studied nsPEF application in preclinical studies. Various research groups have previously confirmed through *in vivo* and *in vitro* experiments that nsPEF can ablate tumor close to the blood vessels causing cell death via multiple mechanisms^[^28,29^]^. Here, we conducted the first-in-human trial to evaluate the safety and efficacy of nsPEF in patients with HCC in high-risk locations.

The LTP rates obtained in this study are similar to those reported following RFA in prospective clinical trials^[^30–32^]^. However, the tumors treated in the present study were in high-risk locations, which can affect RFA treatment outcomes. Further analysis revealed the estimated 3-year cumulative incidences of LTP for perivascular and subcapsular tumors were 13.6% and 17.5%, respectively. Kang *et al* found the 3-year cumulative incidences of the LTP for perivascular tumors in RFA group was 16%. Zheng *et al*^[^33^]^ reported 3-year cumulative LTP rate for subcapsular HCC treated by RFA of 24.6%. Thus, the nsPEF treatment administered in this study achieved significantly better local control and safety of HCC with high-risk localization.

Multivariate analysis demonstrated that maximum tumor diameter served as an independent predictive factor for both LTP and initial complete ablation rate of nsPEF, aligning with prior findings in IRE research. This phenomenon primarily stems from the technical challenges inherent in achieving comprehensive electric field coverage for larger tumors. Specifically, nsPEF and related electroporation-based therapies require meticulous placement of 2–6 electrode needles to generate overlapping electric fields that fully envelop the tumor. Therefore, pulsed electric field therapy mandates a dual-phase protocol: (1) meticulous preoperative planning with three-dimensional imaging guidance to optimize electrode placement; (2) comprehensive postprocedural verification about complete ablation using contrast-enhanced CT/MRI. Furthermore, our trial focused on nsPEF as monotherapy, the preserved tumor antigenic profile observed in preclinical models suggests significant potential for synergizing with immune checkpoint inhibitors. This hypothesis is biologically plausible given nsPEF’s unique capacity to induce immunogenic cell death, which needs to be further verified in clinical trials.

While the study demonstrated a relatively high overall recurrence rate, the 3-year OS reached 91.0% with distinct recurrence patterns: 71.3% of recurrences manifested as new intrahepatic lesions or distant metastases outside the ablation zone. This distribution may be associated with the “field cancerization” effect in cirrhotic livers (78.1%), where the underlying carcinogenic microenvironment promotes multicentric recurrence. Notably, the minimally invasive nature of nsPEF therapy enabled repeated interventions, with the cohort undergoing an average of 2.3 ablative sessions for intrahepatic recurrences. This re-treatability likely constitutes a crucial mechanism sustaining favorable survival outcomes despite frequent recurrence events.

The rate of treatment-related grade 3–4 AE occurrence in this study is similar to rates reported after the thermal ablation of hepatic tumors located in the easily accessible peripheral region (3.5%–7.3%)^[^34,35^]^. Verloh *et al*^[^36^]^ found that complication rates after the thermal (MWA and RFA) and nonthermal (IRE) ablation of malignant liver tumors were comparable, despite the larger number of punctures and lack of track cauterization in IRE. However, direct comparison with our findings is not possible because all tumors included in the present study were close to important structures and thus considered anatomically unsuitable for thermal ablation. No thermal damage to the main bile duct or hepatic vascular structures was observed in this study. Our findings demonstrate that nsPEF treatment is safe in cases characterized by a high risk of thermal damage to neighboring critical structures. The most common side effects in the present study were transient increases in the ALT and AST levels, which had resolved at 1 month after the procedure in the majority of cases. Froud *et al*^[^37^]^ reported extreme increases in transaminase levels within 24 h after IRE in 74.1% of cases, with resolution occurring in 95% of patients. Although dramatic elevations of liver enzymes can occur after pulsed field ablation, most are safe and self-limiting. No sign of liver failure was observed in the present study, and preexisting liver dysfunction was not a contraindication for ablation. However, regular postoperative follow-up should be performed after nsPEF treatment.

Two major complications (upper gastrointestinal bleeding and aspiration pneumonia) occurred under anesthesia in this study. Although, compared with microsecond-scale pulsed field ablation (IRE), nsPEF can minimize their efficacy to stimulate neurons and cardiomyocytes. By far, the nsPEF procedures necessitate the need for general anesthesia and complete muscle relaxation, which increase the additional surgical risks and costs. Looking forward to the next-generation pulsed field ablation technology to conquer technical barriers, and the nsPEF procedure can be conducted under local anesthesia in an outpatient setting.

In recent years, some new alternative treatment methods for thermal ablation have also emerged. In clinical practice, TACE is used as an alternative treatment when RFA is contraindicated due to structural issues. However, the efficacy of TACE depends on the arterial supply, and incomplete response may occur if there is more than a single supply and incomplete embolization^[^38^]^. Recently, radiation treatments such as yttrium 90 (^90^Y) radioembolization and external-beam radiation therapy have emerged as alternative treatments for nonresectable and non–percutaneously ablatable HCC^[^32^]^. However, indications for this technique remain restrained due to its hepatocellular and digestive toxicity. Prospective comparative studies evaluating nsPEF against contemporary locoregional therapies are imperative to delineate their comparative therapeutic niches. Ultimately, reasonable HCC management requires multidisciplinary evaluation balancing tumor biology, anatomical constraints, and patient-specific comorbidities, preference of surgeons, and selection of patients^[^39^]^.

The present study has several limitations. First, being a prospective single-arm study without a control group could lead to selection bias and hinder the assessment of nsPEF’s superiority over other treatment options such as TACE, TARE, or SBRT. Well-designed randomized controlled trials or comparative propensity-scored matched studies are required to further demonstrate the benefits of nsPEF in patients with HCC. Second, the follow-up period was short, and median OS was not achieved. Further long-term studies are needed to provide more definitive evidence of nsPEF’s efficacy. Thirdly, the procedures were performed by experienced interventional radiologists with over 10 years of ablation experience, which may limit the generalizability of the results. More extensive studies involving operators with varying levels of experience are needed to enhance the generalizability of these findings.

## Conclusion

In conclusion, the nsPEF treatment administered in this study is effective and relatively safe for HCC in high-risk locations. The study findings broaden our understanding of electroporation-based medical technologies and providing new minimally invasive therapeutic approaches for HCC suboptimal for thermal ablation. Larger studies are needed to more thoroughly examine the curative potential of the nsPEF treatment.

## Data Availability

The data and materials of this study were available from the corresponding authors.

## Research registration unique identifying number (UIN)

This study has been registered at ClinicalTrials.gov (identifier: NCT04309747)
